# snoRNAs: New players in CKD biomarkers

**DOI:** 10.1016/j.omtn.2026.103013

**Published:** 2026-07-27

**Authors:** Lena Eliasson, Allan Vaag

**Affiliations:** 1 Unit of Islet Cell Exocytosis, Lund University Diabetes Centre (LUDC), Department of Clinical Sciences Malmö, Lund University, Lund, Sweden; 2 Science for Life Laboratory (SciLifeLab), Lund University, Lund, Sweden; 3 Unit of Translational Diabetes Reserach, Lund University Diabetes Centre (LUDC), Department of Clinical Sciences Malmö, Lund University, Lund, Sweden; 4 Steno Diabetes Center Copenhagen, Herlev, Denmark; 5 Department of Endocrinology, Skåne University Hospital, Malmö, Sweden

## Main text

It is a clinical unmet need to identify novel biomarkers to improve diagnosis, prediction and prognosis, as well as response and pharmacodynamics of established and novel therapeutic interventions. Chronic kidney disease (CKD) is a common and severe complication in patients with diabetes, which may not only lead to end-stage kidney disease with a need for transplantation or dialysis, but furthermore is associated with excess morbidity and mortality from cardiovascular disease. While facing these serious consequences, it is important to emphasize that we today are in a much stronger therapeutic position. Intensive blood pressure management, optimized glycemic control, and the introduction of novel highly effective kidney-protective drugs, including sodium-glucose co-transporter 2 (SGLT2) inhibitors, GLP-1 receptor agonists, and mineralocorticoid receptor antagonists, have significantly improved our ability to prevent or delay CKD progression.

For any novel CKD diagnostic or predictive biomarker to be clinically useful, a minimum requirement is that the diagnostic and/or predictive performance outperforms existing diagnostic or predictive risk markers. Current risk stratification already relies on well-established clinical and biochemical parameters, including microalbuminuria; lower eGFR, elevated HbA1c, obesity, insulin resistance, and male gender.^1^ As for the need for novel therapeutic biomarkers in CKD prevention, it would be a quantum leap to have a biomarker that could identify which patients, in terms of CKD prevention, might benefit from medical prevention, including treatment with SGLT2 inhibitors. In this context, it is known that the reno-protective effect of SGLT2 inhibitor therapy cannot be attributed solely to reductions in either blood glucose or blood pressure, and that as-yet-unknown protective mechanisms likely play a major role.

The study by de Klerk et al.^2^ in the June issue of *Molecular Therapy Nucleic Acids* represents a comprehensive and state-of-the-art explorative clinical experimental investigation into circulating small noncoding RNAs (sncRNAs) as potential CKD prognostic or therapeutic biomarkers. Briefly, the investigators analyzed differential expression of a large panel of plasma sncRNAs in 263 individuals with type 2 diabetes from the Hoorn Diabetes Care System (DCS) cohort who were free of CKD at baseline. These individuals were followed for an average of 9 years during which, 141 patients developed CKD. To this end, sncRNA profiling and differential expression analysis was also performed before and after SGLT2 inhibitor treatment in 65 patients. The study identified 11 sncRNAs associated with incident CKD, one sncRNA associated with reduced kidney function (eGFR <60 mL/min/1.73 m^2^), and 31 sncRNAs associated with albuminuria. However, the patients who later developed overt CKD already at baseline showed lower eGFR and higher uACR compared with patients who remained free of CKD. To this end, the magnitudes of differences in sncRNA levels were limited, and after adjustment for known baseline CKD risk factors, all associations with incident CKD were attenuated or disappeared. Following SGLT2 inhibitor treatment, nominal changes in expression levels of 34 sncRNAs was found, but none of these changes remained statistically significant after correction for multiple testing.

Although microRNAs (miRNAs) have received most attention, other sncRNAs, including small nucleolar RNAs (snoRNAs) are emerging as promising biomarker candidates. Traditionally viewed as housekeeping RNAs, snoRNAs are now increasingly recognized for broader regulatory functions and their ability to generate smaller RNA fragments with gene-regulatory roles.^3^ Interestingly, among the identified differentially expressed sncRNAs, the authors highlight the snoRNA, SNORD105B, which in the base model was downregulated in participants with type 2 diabetes who later developed CKD and furthermore associated with albuminuria in both the base and fully adjusted models.^2^ To this end, SNORD105B was nominally significantly upregulated following SGLT2 inhibitor treatment, all together representing a target worth exploring more in other and larger studies, both with respect to its potential as CKD and/or SGLT2-inhibitor biomarker, as well as its potential involvement in the underlying CKD pathophysiological disease mechanisms.

The study idea by de Klerk et al. was well-argued, logical and clinical important, and the study was by all standards well-planned and executed by an experienced team of investigators. Yet, the final study conclusion ends-up being somewhat vague and open to interpretations, which in turn nicely illustrates the contemporary difficulties in using omics technologies to identify novel biomarkers and/or disease mechanisms, as well as the often neglected very high bar for identification of novel clinical useful biomarkers. Regarding the dimension of identifying novel clinically useful CKD diagnostic, predictive, and/or prognostic biomarkers, an absolute requirement is that these needs to be superior to all already known clinical as well as biochemical markers of CKD risk and/or diagnosis. As already mentioned, none of the investigated sncRNAs fulfilled these criteria. Whereas this may not be enough to exclude the here investigated snoRNAs as future potentially clinical useful CKD biomarkers, it does reduce our overall enthusiasm and hope for this to be the case. Likewise, the fact that none of the snoRNAs changed significantly in response to SGLT2-inhibitor treatment after adjustment for multiple testing may not raise our immediate expectation of any of these to represent novel target engagement SGLT2-inhibitor treatment biomarkers.

On the more positive side, however, the study has indeed raised our attention of snoRNAs in general, and of SNORD105B in particular, potentially to play even very important roles in the molecular mechanisms underlying the development of CKD. SnoRNAs are increasingly being detected in circulation, particularly within extracellular vesicles (EVs), and several studies have shown that specific snoRNAs are differentially expressed in serum or plasma in diseases including cancer and cardiovascular conditions, supporting their potential use as non-invasive biomarkers.^3^ Compared with miRNAs, the biomarker field for snoRNAs is still in its early stages but is rapidly evolving. SnoRNAs may offer certain advantages, including the ability to provide complementary information to established regulatory RNA pathways. Nevertheless, miRNAs currently remain more mature biomarkers, supported by larger datasets and more standardized methodologies. Importantly, however, no miRNA has yet been adopted as a routine clinical biomarker for diabetes complications. Studies have often produced inconsistent results, with limited reproducibility across populations. For instance, miR-126 has been proposed as a biomarker in several studies,^4^ yet failed to predict incident diabetes or cardiovascular outcomes in larger cohorts, where alternative candidates such as miR-483-5p emerged instead.^5^ These discrepancies highlight the importance of harmonization in study design, larger sample sizes, and rigorous validation.

Finally, it is important to emphasize that it is too early to dismiss SNORD105B, or any other sncRNA as potential biomarkers of therapeutic response. Here, the direction of changes with levels being lower at baseline in patients who later developed CKD, along with levels increasing with SGLT2-inhibitor treatment, may appear encouraging. Notably, it is important to emphasize that we still do not know what exact molecular mechanisms that are responsible for the undisputed kidney protection conferred by SGLT2-inhibitor treatment. However, what we do know is that this effect is neither explained by blood glucose nor blood pressure reducing effects of SGLT2-inhibitor treatment.^6^ Accordingly, conventional clinical blood pressure or HbA1c markers do not help identifying responders to kidney protection by SGLT2-inhibitor treatment, why there is an urgent need—and potentially relatively lower hanging fruit—for sncRNAs in general and especially the snoRNA SNORD105B to fill this important clinical biomarker gap. Importantly, future efforts should likely focus on developing panels of complementary biomarkers rather than relying on a single marker, as such approaches may provide greater predictive power and clinical utility. We, therefore, impatiently and with great interest await further studies addressing this question.

## Declaration of interests

The authors declare no competing interests.
